# Ammonia-Oxidizing Archaea (AOA) Play with Ammonia-Oxidizing Bacteria (AOB) in Nitrogen Removal from Wastewater

**DOI:** 10.1155/2018/8429145

**Published:** 2018-09-13

**Authors:** Zhixuan Yin, Xuejun Bi, Chenlu Xu

**Affiliations:** ^1^Qingdao University of Technology, Qingdao 266033, China; ^2^State and Local Joint Engineering Research Center of Municipal Wastewater Treatment and Resource Recycling, Qingdao 266033, China

## Abstract

An increase in the number of publications in recent years indicates that besides ammonia-oxidizing bacteria (AOB), ammonia-oxidizing archaea (AOA) may play an important role in nitrogen removal from wastewater, gaining wide attention in the wastewater engineering field. This paper reviews the current knowledge on AOA and AOB involved in wastewater treatment systems and summarises the environmental factors affecting AOA and AOB. Current findings reveal that AOA have stronger environmental adaptability compared with AOB under extreme environmental conditions (such as low temperature and low oxygen level). However, there is still little information on the cooperation and competition relationship between AOA and AOB, and other microbes related to nitrogen removal, which needs further exploration. Furthermore, future studies are proposed to develop novel nitrogen removal processes dominated by AOA by parameter optimization.

## 1. Introduction

Nitrogen-containing pollutants are considered one of the most common environmental pollutants in various types of wastewater, and they are an important pollution factor that causes eutrophication. The conventional biological system for nitrogen removal from wastewater is usually through the biological oxidation of ammonia and organic nitrogen (nitrification) and the biological reduction of the oxidation products, that is, nitrate (denitrification). From the viewpoint of microbial transformation of nitrogen, the nitrification process includes ammonia oxidation (NH_3_-N → NO_2_^−^-N) and nitrite oxidation (NO_2_^−^-N → NO_3_^−^-N). As the rate-limiting step of the nitrification, ammonia oxidation is the key process for biological nitrogen removal from wastewater, thus attracting wide attention from researchers.

In the past 100 years, ammonia-oxidizing bacteria (AOB) were considered as the dominant microorganism in the ammonia oxidation process [1]. With the development of molecular biology techniques in recent years, it had been found that the *amoA* gene, a kind of indicative gene of ammonia oxidation, exists in large numbers of archaea distributed in the marine environment, proving that archaea also have the capacity of ammonia oxidation at the physiological metabolic level [2]. Hereafter, ammonia oxidations conducted by archaea were widely found in hot springs, soils, oceans, sediments, and wetlands and these archaea were formally known as ammonia-oxidizing archaea (AOA) in subsequent studies [3–5]. In addition, a large number of studies have reported that the AOA abundance and the archaeal *amoA* gene abundance are significantly higher than that of AOB in farmland soils, river sediments, and oceans [6], indicating that AOA are the main driver of ammonia oxidation in these habitats and play a more important role in the global nitrogen cycle.

## 2. Cell Structure and Metabolism Physiology of AOA

The cell volumes of most AOA are 10 to 100 times smaller than those of known AOB. This has implicated that the ammonia oxidation rates per cell for *Nitrosopumilus maritimus* SCM1 (AOA) were reported to be 10-fold lower than those of AOB [7]. Thus, the individual contributions of AOA and AOB to ammonia oxidation should be identified by considering not only the relative abundance of cell counting but also activity-correlated analyses [8]. The tetraether lipid-based membranes of AOA cells make it less permeable to ion than AOB membranes, thus resulting in the reduction in the amount of futile ion cycling and lower levels of maintenance energy relative to AOB, offering the advantages of their adaption to extreme environments [9]. In addition, according to cryoelectron tomography data, the cells of *Nitrosopumilus maritimus* SCM1 in exponential growth harbor ~1000 ribosomes per ~0.023 *μ*m^3^ cell volume [10]. The high numbers of ribosomes of AOA offer organisms the ability to respond quickly to changing environmental conditions (e.g., fluctuating ammonia levels). This is consistent with the observations that most archaea, in contrast to bacteria, are highly adapted to energy-stressed environments [9]. Available data on the stability of mRNAs, ammonia monooxygenase (AMO), and ribosomal proteins of AOA are still lacking but could be essential in understanding the ecological adaptations of AOA compared to AOB.

**Figure 1:**
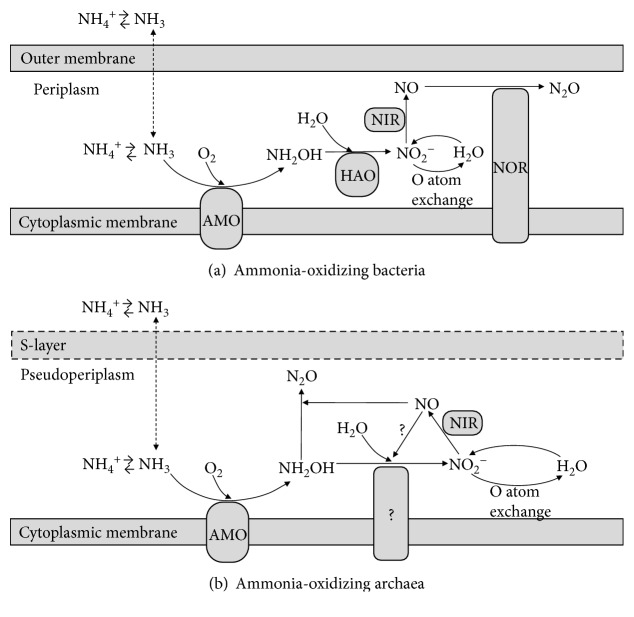
Schematic illustration of ammonia oxidation pathways in ammonia-oxidizing bacteria (a) and archaea (b). The figure is reproduced from Kozlowski et al. and Nishizawa et al. [66, 67]. Abbreviations: HAO, hydroxylamine dehydrogenase; NIR, nitrite reductase; NOR, nitric oxide reductase.

It is generally accepted that not NH_4_^+^ but NH_3_ is the substrate for bacterial AMO [11], while the true substrate for archaeal AMO remains to be elucidated. As shown in Figure 1, in AOB, the membrane-associated AMO catalyzes the aerobic oxidation of NH_3_ to hydroxylamine (NH_2_OH) which is subsequently oxidized to NO_2_^−^ by the periplasmic hydroxylamine oxidoreductase (HAO) [12]. Without the discovery of the HAO homologue, enzymes for the detoxification of NH_2_OH, or cytochrome *c* in any AOA genome, it is unclear whether archaeal AMO catalyzes the same reaction as AOB [13, 14]. Either archaeal AMO reaction or unidentified enzyme substitutes for HAO in AOA might yield a different product [14]. It was suggested that nitroxyl hydride (HNO) might be generated by archaeal AMO, which could be subsequently oxidized to NO_2_^−^ via nitroxyl oxidoreductase (NxOR) [14]. The activation of O_2_ for the monooxygenase reaction could also be achieved by nitric oxide (NO), the reaction product of nitrite reductase (NIR), which would result in N_2_ gas production [2]. It was also reported that archaeal *nirK* (encoding copper-dependent NIR) genes are expressed under aerobic conditions [15, 16], suggesting a different behaviour of these enzymes in AOA compared to the bacterial counterparts. Furthermore, the lack of cytochrome *c* proteins and the existence of numerous genes encoding copper-containing proteins (multicopper oxidases and plastocyanin-like domain proteins) in AOA suggest a different electron transport mechanism [14] from that of the highly iron-heme-dependent AOB [17, 18]. A copper-based biochemistry would help to explain the ecological success of marine AOA (compared to AOB), because dissolved copper concentrations are generally an order of magnitude higher than those of iron in seawater [10].

## 3. The Discovery of AOA in Wastewater Treatment System

The first report on AOA in wastewater treatment systems was reported in 2006. Park et al. [19] detected the archaeal *amoA* gene from the activated sludge in nitrification tanks of five wastewater treatment plants in the United States through the polymerase chain reaction (PCR) method. However, due to limited technical means at that time, it was difficult to obtain quantitative data of the absolute abundance of AOA. In 2009, Wells et al. [20] used quantitative PCR to detect AOA in a wastewater treatment system for the first time. Since then, the researchers focused their attention on the comparison of AOA and AOB abundance in the wastewater system for nitrogen removal, as shown in Table 1. Some researchers found that the abundance of AOA was higher than that of AOB in domestic wastewater treatment systems [21–25], whereas the situation was reversed in the systems for industrial wastewater treatment [23–25]. However, Gao et al. [26, 27] found that the abundance of AOB was approximately 3 orders of magnitude higher than that of AOA in the investigation of 8 wastewater treatment systems (including industrial wastewater and domestic wastewater) in Beijing. Muβmann et al. [28] found high abundance of AOA in four industrial wastewater treatment systems, and even the abundance of AOA in one of the systems was 4 orders of magnitude higher than AOB. Zhang et al. [29] showed that high concentrations of spiramycin caused a significant increase in the relative abundance of AOA in pharmaceutical wastewater treatment systems.

In recent years, AOA have been successfully cultivated and enriched in pure medium [30–33], but there is still no information on the enrichment of AOA in the actual wastewater nitrogen removal system. Using inorganic medium, Sonthiphand and Limpiyakorn [34] had attempted to enrich ammonia-oxidizing microorganisms in activated sludge which contained a nearly equal number of archaeal *amoA* genes to bacterial *amoA* genes, but AOA gradually disappeared from the ammonia-oxidizing consortiums in all reactors with the prolongation of cultivation time. Compared with suspended floc activated sludge, stable ecological conditions of attached biofilm provide a habitat for more microbes especially with long generation. Roy et al. [35] found that AOA outnumber AOB and contribute to ammonia oxidation in the biofilm samples of trickling filter and moving bed bioreactor treating municipal wastewater, with the abundance of the archaeal *amoA* gene 2-3 orders of magnitude higher than that of the bacterial *amoA* gene. Chen et al. [36] also had the same observation in the biofilm in biological aerated filters for municipal wastewater treatment, and a single AOA strain was enriched from the filtering materials using synthetic medium [37].

Based on the reviewed literature, the distribution of AOA and AOB in different wastewater treatment systems is still unclear, and the differences in the research results may be affected by the characteristics of treated wastewater (ammonia level, organic loading) and process operating parameters (temperature, dissolved oxygen (DO) concentration) [38].

## 4. Environmental Factors Affecting AOA and AOB

### 4.1. Ammonia Level

As a common substrate (nitrogen source) of AOA and AOB, the concentration of ammonia in the environment significantly influences the growth of these two kinds of ammonia-oxidizing microorganisms. AOA have a higher affinity for ammonia than AOB [7, 21], resulting in lower inhibitory concentration for AOA. Exposed in a higher ammonia concentration, AOA might face the suppressed situation earlier than AOB. Sauder et al. [39] demonstrated that the amount of AOA *amoA* gene was reduced with the increase in the ammonia concentration in the rotating biological contactors of a municipal wastewater treatment plant, indicating that AOA were suitable for low ammonia level. According to Gao et al. [40], AOB were more competitive than AOA under high concentrations of ammonia, and the higher the ammonia concentration was, the higher the AOB abundance was [28]. There was also no big difference in the abundance of AOA at different ammonia nitrogen levels (14, 56, and 140 mg N/L) [28]. Ye and Zhang [41] observed that in the nitrification tank for salty wastewater treatment, when the concentration of ammonia increased from 200 mg/L to 300 mg/L, the abundance of AOA was considerably reduced but the abundance of AOB remained stable. In addition, in the landfill leachate treatment system with a high ammonia concentration (2180 ± 611 mg N/L), the ammonia oxidation process was dominated by AOB [42]. It could be concluded that the level of ammonia which was affected by the types of wastewater could result in the differences in the microbial community structure of AOA and AOB.

### 4.2. Organic Loading

Organic matter objectively affects the growth of ammonia-oxidizing microorganisms. AOB are recognized as autotrophic microorganisms, while it is not clear whether AOA are strictly autotrophic or mixotrophic. Some studies have reported that the presence of organic substances had a significant inhibitory effect on the growth of some certain AOA strains such as *Nitrosopumilus maritimus* SCM1 and *Nitrosocaldus yellowstonii* [43, 44]. The latest study found that the addition of organic substances could promote the growth of AOA strains PS0 and HCA1, showing their characteristics of mixotrophic growth [45]. It had also been proved using genome sequencing that some AOA strains had two different carbon utilization mechanisms: 3-hydroxypropionic acid/4-hydroxybutyric acid cycle (autotrophic metabolism) and tricarboxylic acid cycle (heterotrophic metabolism), indicating that these AOA strains had the potential for autotrophic and heterotrophic metabolism [30, 46]. Compared with AOB, AOA may have more complex metabolic pathways and may show different metabolic characteristics under different carbon source conditions, resulting in changes in ammonia oxidation capacity of AOA and AOB.

### 4.3. Temperature

The effect of temperature on ammonia-oxidizing microorganisms is mainly manifested in the effect on the activity of ammonia monooxygenase [47]. The currently found AOB belong to mesophiles, while the range of adaptation temperature of AOA is very large. It could be observed that active ammonia oxidations by AOA occur at 0.2 °C in the deep water region of the North Japan Sea and at 74 °C in the hot spring in Yellowstone National Park [44, 48]. He et al. [49] found that the dominant ammonia oxidation microorganisms in the sediments near the Rushan Bay of Shandong Peninsula were AOB during the summer (water temperature = 21–25 °C) while AOA in the winter (water temperature = 3-4 °C). Niu et al. [50] found that in the biological activated carbon filtration system for drinking water purification, the AOB *aomA* gene abundance decreased significantly in winter (water temperature = 4.6–5.5 °C) compared with that in summer (water temperature = 17.7–28.6 °C), while the AOA gene abundance changed little. Sims et al. [51] also observed that AOB were more sensitive to low temperatures than AOA in the constructed wetland system for wastewater treatment. The adaptation ability of AOA to temperature changes is inseparable with the special structure of glycerol ether in the cell membrane, thus making the activity of ammonia monooxygenase relatively less affected by temperature and endowing AOA with a competitive advantage under extreme temperature conditions.

### 4.4. Oxygen

Oxygen is a necessary reaction substrate of the nitrification process. Due to the difference in the affinity of nitrifying microbes for oxygen (AOA > AOB > NOB (nitrite-oxidizing bacteria)), the oxygen concentration will affect the nitrification process. High oxygen affinity makes AOA more competitive than AOB in hypoxic environments such as deep oceans, deep soils, and sediments [7, 52]. Park et al. [19] detected large amounts of AOA with low dissolved oxygen level (<0.2 mg/L) in the outer ditch of an Orbal oxidation ditch, and found that simultaneous nitrification and denitrification occurred in the outer ditch at the same time [53]. Li et al. [54] also predicted that AOA and heterotrophic denitrifying bacteria could be coupled in a single reactor by reducing the aeration pressure to inhibit the activity of NOB, and nitrogen could be removed by shortcut simultaneous nitrification and denitrification. In addition, using real-time quantitative PCR, Yapsakli et al. [42] detected the coexistence of AOB, NOB, AOA, and anaerobic ammonium oxidation (anammox) bacteria at low dissolved oxygen (DO = 0.3–1.5 mg/L) in the system for landfill leachate treatment. Establishing a mathematical model, Liu et al. [55, 56] predicted that in a wide ammonia nitrogen concentration range (30–500 mg/L), with less oxygen consumption and stronger inhibitory effect on NOB activity, autotrophic nitrogen removal by coupled AOA nitritation with anammox was more effective than coupled AOB with anammox. Nitrogen removal by the cooperative AOA, AOB, and denitrifying bacteria or anammox bacteria could be achieved through the regulation of dissolved oxygen level to optimize the community structure. It is also expected to provide new ideas for the development of wastewater nitrogen removal process with high efficiency and low consumption [36, 57].

### 4.5. pH

It was reported that the pH range of AOA strain SAT1 enriched from activated sludge was 5.0 to 7.0, with the optimum pH at 6.0, indicating that the strain SAT1 was neutrophilic [31]. The ammonia bioavailability can be reduced by the protonation of ammonia when pH decreases, which might be more favourable for the growth of AOA from the perspective of substrate utilization. Recent studies had provided evidence that ammonia oxidation in acidic soils was dominated by AOA, whereas AOB had difficulty surviving at low pH values and were mainly responsible for nitrification in alkaline soils [58–62]. However, it was also reported that alkaline soil was also suitable for the growth of *Candidatus* Nitrosotalea devanaterra (AOA) [63] which showed strong adaptability to pH variation. Until now, the differences in the relative contributions of these two groups of ammonia oxidation microorganisms affected by environmental pH remain a topic of debate. There is also little information concerning the effects of pH on the distribution of AOA and AOB in wastewater treatment systems. However, the AOA strain with strong adaptability to pH changes provides the possibility of its application in wastewater treatment systems with acidic influent.

**Figure 2:**
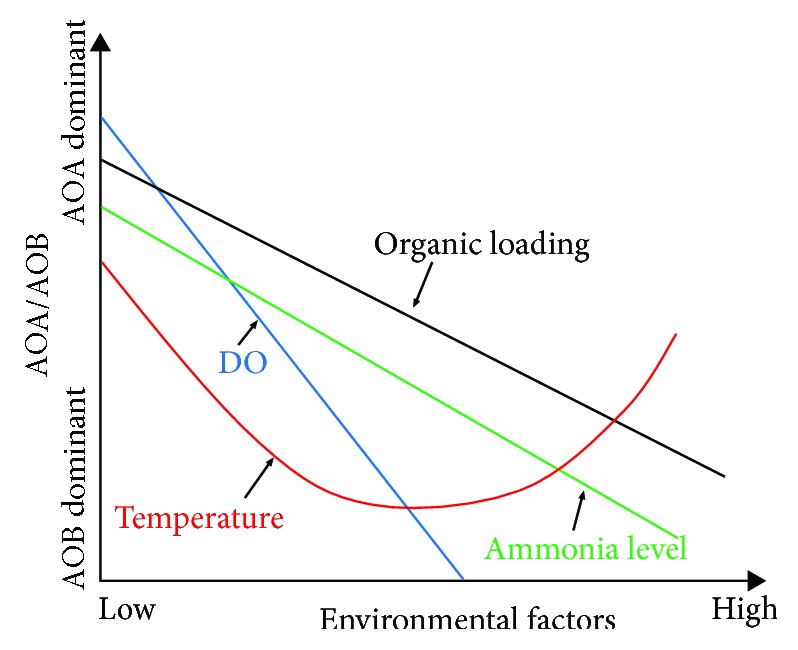
The proposed AOA/AOB in response to the varying environmental factors (ammonia, organic loading, oxygen level, and temperature) (based in part on Guo et al. [68]).

Based on the literature review above, AOA/AOB in response to the varying environmental factors including ammonia, organic loading, oxygen level, and temperature is proposed in Figure 2. AOA would be dominant over AOB in low ammonium and/or low DO and/or low organic loading environments. AOA would also be more active than AOB when they are exposed to extreme high/low temperatures. In addition, compared with AOB, AOA would be dominant in salinity-containing wastewater [64, 65].

## 5. Recommendations for Further Study Associated with AOA

Since the discovery of AOA in wastewater treatment plant bioreactors in 2006 [19], AOA have been recognized as potential ammonia oxidizers involved in nitrogen removal from wastewater. The current available information indicates that knowledge of these microorganisms in engineered systems is still at a primary stage. Challenges for practical application include the complexity of wastewater, the uncertainty of operational parameters affecting the activity and functions of AOA, and the limitations of the techniques available. Combined microbiological and engineering points of view are required in the future study. According to the latest literature reviewed, the following further studies were recommended:

1. Compared with AOB, AOA behaved more active in extreme environments. Therefore, AOA are expected to be effectively enriched and cultured under low temperature conditions or low dissolved oxygen level in wastewater treatment systems (probably in biofilm systems), thus solving the problem of poor nitrification that often happens in wastewater treatment plants in cold regions and providing a new breakthrough for an effective nitrification process.
2. Although the prediction results of a mathematical model increase the possibility of the development of novel nitrogen removal processes dominated by AOA coupled with denitrifying bacteria or anammox bacteria [55, 56], the structure of the ammonia oxidation functional microbes still needs to be further studied in the actual wastewater nitrogen removal system. The optimization of process parameters is also necessary to achieve effective nitrogen removal.
3. The variations in the population structure of microorganisms (AOA, AOB, NOB, anammox bacteria, and denitrifying bacteria) and their contributions to the nitrogen removal process in actual wastewater treatment systems under different environmental conditions need to be investigated to explain the coexistence, coordination, and competition mechanisms among the microbes associated with the nitrogen removal function.

## 6. Conclusions

The discovery of AOA breaks the traditional view for the past 100 years that ammonia oxidation is only conducted by AOB, improving the knowledge of the global nitrogen cycle. AOA also appear to play an important role in nitrogen removal from wastewater. Hence, the nitrogen cycle in a wastewater treatment system needs reevaluation. The collaborative, competitive, and inhibitive relationships in microbial communities need further exploration in actual wastewater nitrogen removal systems. The ammonia-oxidizing microorganisms are affected by various environmental conditions, and AOA have stronger environmental adaptability than AOB, which provides the possibility for the development of novel nitrogen removal processes with ammonia oxidation dominated by AOA under extreme environmental conditions (such as low temperature and low oxygen level).

## Figures and Tables

**Table 1:** Comparison of AOA and AOB in different wastewater treatment systems.

Biomass samples	Influent			Process parameter		AOA *amoA* gene abundance	AOB *amoA* gene abundance	AOA?AOB	Reference
	Wastewater type	Ammonia level (mg N/L)	COD (mg/L)	Temperature (°C)	DO (mg O_2_/L)				
AS^a^	Municipal wastewater	14–33	116–233	18.2–25.4	3.08–4.50	8 × 10^1^–2 × 10^3^ copies mL^−1^ sludge	1.2 × 10^6^–4.1 × 10^6^ copies mL^−1^ sludge	AOA < AOB	[20]
AS	Municipal wastewater	5.4–38.6	24.8–152.0	N/A	0.5–3.25	3.28 × 10^4^ ± 1.74 × 10^4^–2.23 × 10^8^ ± 1.92 × 10^8^ copies mL^−1^ sludge	8.05 × 10^3^ ± 5.20 × 10^3^–5.72 × 10^6^ ± 5.69 × 10^5^ copies mL^−1^ sludge	AOA > AOB	[22]
AS	Municipal wastewater	16.3–76.6	115–580	N/A	N/A	6.3 × 10^5^–4.5 × 10^6^ copies g^−1^ sludge	7.2 × 10^3^–1.7 × 10^5^ copies g^−1^ sludge	AOA > AOB	[23]
AS	Municipal wastewater	5.6–11.0	23.4–68.0	N/A	N/A	1.05 × 10^5^ ± 6.74 × 10^4^–7.48 × 10^8^ ± 2.08 × 10^8^ copies mL^−1^ sludge	3.73 × 10^5^ ± 3.07 × 10^5^–9.05 × 10^7^ ± 2.77 × 10^7^ copies mL^−1^ sludge	AOA > AOB	[24]
AS	Municipal wastewater	14–58	154–603	N/A	0.2-3.5	9.38 × 10^2^ ± 4.74 × 10^1^–1.11 × 10^6^ ± 1.46 × 10^6^ copies g^−1^ sludge	1.50 × 10^5^ ± 6.90 × 10^4^–3.32 × 10^8^ ± 6.10 × 10^7^ copies g^−1^ sludge	AOA < AOB	[26]
AS	Municipal wastewater	15.9	174	30	1.2	1.11 × 10^3^ ± 3.02 × 10^1^–2.35 × 10^3^ ± 7.34 × 10^1^ copies ng^−1^ DNA	6.35 × 10^1^ ± 2.3–1.76 × 10^2^ ± 1.56 × 10^1^ copies ng^−1^ DNA	AOA > AOB	[38]
AS	Municipal wastewater	35.8	336	16	1.7	<LOD^b^	1.36 × 10^3^ ± 3.68 × 10^1^–2.71 × 10^4^ ± 1.35 × 10^4^ copies ng^−1^ DNA	AOA < AOB	[38]
AS	Municipal wastewater	15.9	110	22	1.4	<LOD	3.69 × 10^4^ ± 1.5 × 10^3^ copies ng^−1^ DNA	AOA < AOB	[38]
AS	Municipal wastewater	18.3	100	N/A	N/A	23–39 copies ng^−1^ DNA	16–220 copies ng^−1^ DNA	AOA < AOB	[64]
AS	Municipal wastewater	N/A	N/A	13–23	N/A	1.6 × 10^2^–1.9 × 10^2^ copies ng^−1^ DNA	1.1 × 10^3^ copies ng^−1^ DNA	AOA < AOB	[69]
AS	Municipal wastewater	N/A	N/A	10–18	N/A	1.0 × 10^2^–4.0 × 10^2^ copies ng^−1^ DNA	1.1 × 10^3^–1.3 × 10^3^ copies ng^−1^ DNA	AOA < AOB	[69]
AS	Municipal/industrial wastewater	20.5–474.8	365.2–2508.7	N/A	1.5–7.5	<LOD-1.9 × 10^7^ copies g^−1^ sludge	4.625 × 10^4^–9.99 × 10^9^ copies g^−1^ sludge	AOA < AOB	[27]
AS	Industrial wastewater	35.2–262.0	524–2730	N/A	N/A	5.7 × 10^3^–9.9 × 10^3^ copies g^−1^ sludge	2.6 × 10^7^–3.6 × 10^9^ copies g^−1^ sludge	AOA < AOB	[23]
AS	Industrial wastewater	36.1–422.3	192–1410	N/A	N/A	<LOD	2.78 × 10^6^ ± 1.32 × 10^6^–4.25 × 10^7^ ± 9.65 × 10^6^ copies mL^−1^ sludge	AOA < AOB	[24]
AS	Spiramycin production wastewater	249	4575	N/A	N/A	1.72 × 10^5^ ± 3.02 × 10^5^ copies ng^−1^ DNA	3.25 × 10^4^ ± 3.17 × 10^2^ copies ng^−1^ DNA	AOA > AOB	[29]
AS	Oxytetracycline production wastewater	164	3200	22	N/A	3.6 × 10^1^ ± 3.0 × 10^1^ copies ng^−1^ DNA	3.9 × 10^4^ ± 1.94 × 10^3^ copies ng^−1^ DNA	AOA < AOB	[29]
AS	Landfill leachates	2180 ± 611	5565 ± 3397	N/A	0.3–2.5	<LOD-1.1 × 10^4^ ± 2.0 × 10^2^ cells in extracted DNA	2.1 × 10^3^ ± 4.0 × 10^1^–1.3 × 10^5^ ± 1.0 × 10^3^ cells in extracted DNA	AOA < AOB	[42]
Biofilm	Municipal wastewater	9.8	104	N/A	N/A	6.0 × 10^5^ copies g^−1^ sludge	3.6 × 10^4^ copies g^−1^ sludge	AOA > AOB	[23]
Biofilm	Municipal wastewater	0.3–7.2	N/A	10–22	2–5	2.2 ± 0.3–7.8 ± 0.9 copies *μ*L^−1^ DNA	9.2 ± 0.7–128.0 ± 4.0 copies μL^−1^ DNA	AOA < AOB	[35]
Biofilm	Municipal wastewater	N/A	N/A	10–22	2–5	4.5 × 10^5^ ± 0.1 × 10^5^–1.9 × 10^6^ ± 0.3 × 10^6^ copies μL^−1^ DNA	4.5 × 10^3^ ± 0.1 × 10^3^–1.1 × 10^4^ ± 0.1 × 10^4^ copies μL^−1^ DNA	AOA > AOB	[35]
Biofilm	Municipal wastewater	2.7–11.7	43–121	10–22	5	2.2 × 10^6^ ± 0.1 × 10^6^–1.0 × 10^7^ ± 0.1 × 10^7^ copies μL^−1^ DNA	3.4 × 10^4^ ± 0.3 × 10^4^–1.0 × 10^5^ ± 0.3 × 10^5^ copies μL^−1^ DNA	AOA > AOB	[35]
Biofilm	Municipal wastewater	10.6	38	23.6–24.0	0.9–4.6	6.32 × 10^3^–3.8 × 10^4^ copies ng^−1^ DNA	20.6–105.2 copies ng^−1^ DNA	AOA > AOB	[36, 37]
Wetland soil	Effluent from WWTP	20–30	45–70	5.5–24	N/A	2.1 × 10^6^ ± 0.2 × 10^6^–1.8 × 10^7^ ± 0.2 × 10^7^ copies g^−1^ soil	1.2 × 10^5^ ± 0.2 × 10^5^–5.2 × 10^7^ ± 0.2 × 10^7^ copies g^−1^ soil	AOA > AOB	[51]

^a^AS, activated sludge. ^b^LOD, limit of detection.
